# Winter activity of tricolored bats in aboveground and subterranean hibernacula in the southeastern USA

**DOI:** 10.1038/s41598-025-97703-y

**Published:** 2025-04-20

**Authors:** Susan C. Loeb, William C. Bridges, Eric A. Winters, Rebecca L. Brown, Jessica R. Anderson, Mack Ferrari, Jordyn R. Upton, Lisa M. Smith, Thomas C. McElroy, Andrew J. Edelman, Christopher T. Cornelison

**Affiliations:** 1 USDA Forest Service, Southern Research Station, Clemson, SC 29634 USA; 2 Department of Mathematical and Statistical Sciences, Clemson University, Clemson, SC 29634 USA; 3 Department of Forestry and Environmental Conservation, Clemson University, Clemson, SC 29634 USA; 4 Biology Program, University of West Georgia, Carrollton, GA 30118 USA; 5 Department of Molecular and Cellular Biology, Kennesaw State University, Kennesaw, GA USA; 6 Fish and Wildlife Research Institute, Florida Fish and Wildlife Conservation Commission, Gainesville, FL 32601 USA; 7 Department of Ecology, Evolution, and Organismal Biology, Kennesaw State University, Kennesaw, GA USA

**Keywords:** Cave, Culvert, Hibernacula, *Perimyotis subflavus*, Torpor, White-nose syndrome, Ecology, Zoology, Ecology

## Abstract

Susceptibility of bats to white-nose syndrome (WNS), a lethal disease caused by the fungus *Pseudogymnoascus destructans* (*Pd*), may be influenced by the amount of activity outside hibernacula during the winter. We tested the effects of hibernaculum type (aboveground or subterranean) and *Pd* status (positive or negative) on winter activity of tricolored bats (*Perimyotis subflavus*) in the southeastern USA along with the effects of ambient temperature, precipitation, and stage of hibernation. We placed acoustic detectors at the entrances of 13 hibernacula (4 aboveground and *Pd*-positive, 4 aboveground and *Pd-*negative, 4 subterranean and *Pd*-positive, and 1 subterranean and *Pd*-negative) during winter 2020–21 and 2021–22. While neither hibernaculum type nor *Pd* status alone predicted probability of activity or levels of activity, these factors interacted with temperature, precipitation, and stage of the hibernation period. Activity increased at a greater rate with temperature and time since the onset of hibernation in aboveground and *Pd*-negative sites and decreased at a faster rate in response to precipitation. Our results suggest that tricolored bats using aboveground hibernacula such as culverts or bridges may be less susceptible to WNS due to greater nighttime activity. However, use of these structures may have other costs such as higher freezing and predation risks.

## Introduction

Many species use extended torpor or hibernation during winter as a means to manage energetic demands resulting from cold temperatures and reduced food availability^1^. During these periods, animals allow their body temperatures to drop towards ambient, thus decreasing their metabolic costs. Bouts of torpor are interrupted by episodic arousals that serve many purposes including ridding the body of accumulated wastes, reactivating the immune system, and alleviating dehydration and sleep deprivation^2,3^. Among bats, these arousal periods are also sometimes characterized by activity outside the hibernaculum to switch roosts, drink, and occasionally forage^4–7^. Little is known about the behavior of bats outside hibernacula during winter, but this information is essential for effective management of winter habitats as well as understanding bats’ responses to stressors such as climate change and disease^7,8^. For example, increased activity during winter due to warming temperatures may represent an ecological trap if resources such as insects or water are not available^9^.

White-nose syndrome (WNS) is a fungal disease that affects bats during winter hibernation^10^. The fungus invades bats’ epidermis, resulting in a series of physiological responses, including increased arousals from torpor, increased blood CO_2_levels, water and electrolyte loss, and hyperventilation^11^. The combined effect of these responses is an increase in energy use and consequently, the depletion of bats’ fat reserves and in many cases, death. The disease was first documented in upstate New York, USA in 2006^12^and was most likely introduced from Europe^13,14^. Since its introduction, the fungus that causes WNS (*Pseudogymnoascus destructans*; hereafter referred to as *Pd*) has spread widely throughout much of the Midwestern, northeastern, and southern regions of the U.S., as well as parts of the western U.S. and up through much of southern Canada (https://whitenosesyndrome.org/where-is-wns). Mortality in some species has resulted in > 90% declines in populations within the affected area^15^.

Most of the mortalities from WNS have occurred within four species: northern long-eared bats (*Myotis septentrionalis*), little brown bats (*M. lucifugus*), Indiana bats (*M. sodalis*), and tricolored bats (*Perimyotis subflavus*)^15^. *Pd *has been found on many other species that do not show diagnostic symptoms of the disease^16^, whereas other species that are infected appear to be far less susceptible to its effects. For example, big brown bats (*Eptesicus fuscus*) show diagnostic signs of the disease but have not exhibited significant population declines^15,17^. Decreased susceptibility of big brown bats to WNS compared to species such as little brown bats may be due to thermoregulatory responses^18^, larger body size and fat reserves^19^, differences in skin fatty acid composition and higher concentrations of fatty acids with anti-fungal properties^20–22^, and higher wing acidity^23^. Rafinesque’s big-eared bats (*Corynorhinus rafinesquii*) and southeastern myotis (*M. austroriparius*) also do not appear to be susceptible to WNS, perhaps due to their shallow torpor patterns and frequent arousals from winter torpor resulting in higher immune function^24,25^. Because *Pd *does not grow when temperatures are > 19.0 °C^26^, bats that exhibit shallow torpor and frequent nighttime activity may be less susceptible to WNS than those that go into prolonged periods of deep torpor because their body temperatures rise above the growth threshold for *Pd *and their immune systems may be more active^27–30^. Thus, knowledge of the torpor and activity patterns of bats throughout winter may help predict the response of bats to *Pd* once the fungus has been transferred to an area.

Tricolored bats are found throughout the eastern USA and are one of the species most severely impacted by WNS^15^. Across much of their range tricolored bats hibernate in subterranean structures such as caves, mines, and tunnels^31^. However, in areas where these structures are not available, such as much of the southern U.S., they roost in culverts, bridges, and trees during winter^32–36^. Tricolored bats that use these aboveground structures can go into deep torpor with torpor bouts lasting up to 15.5 days but they may also leave the roost at night to forage or switch roosts^28^. Arousals from torpor in aboveground hibernacula usually occur around dusk. In contrast, tricolored bats that hibernate in caves and tunnels arouse randomly throughout day and night although there is little evidence that tricolored bats in belowground leave the hibernacula^37^.

**Fig. 1:**
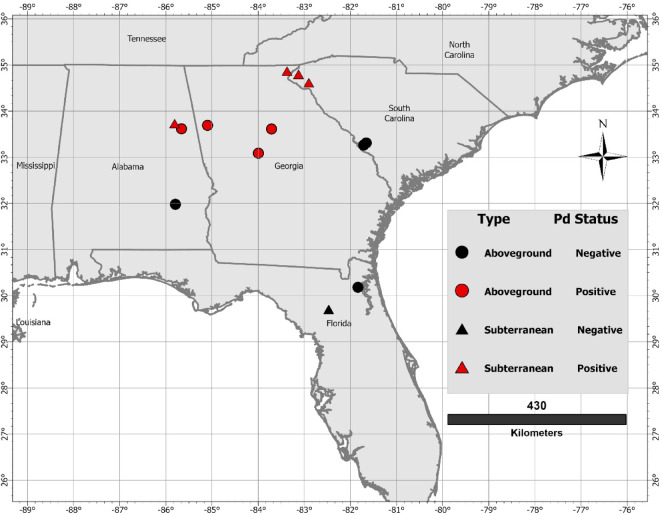
Location of tricolored bat (Perimyotis subflavus) hibernacula monitored for winter activity across the southeastern USA.

 While *Pd* and WNS are prevalent in much of the southeastern U.S., the fungus has not been detected in many areas, particularly along the Atlantic and Gulf Coasts and in Florida (https://whitenosesyndrome.org/where-is-wns). To better understand the potential for morbidity and mortality if the fungus arrives in these areas, we studied activity outside the roost in tricolored bats roosting in subterranean and aboveground hibernacula in four southeastern U.S. states (Fig. 1). Because greater activity, particularly on warmer nights when insects may be active, may decrease bats’ susceptibility to WNS, and bats in aboveground hibernacula may be more active those in subterranean hibernacula, our objective was to test the effects of hibernaculum type (aboveground or subterranean) on activity of tricolored bats outside hibernacula during winter. Although WNS causes bats to arouse more frequently during hibernation and often leave the hibernacula^5,38^, arousals return to pre-WNS frequency during the endemic phase of the disease^39^. Thus, activity outside hibernacula in the endemic phase of the disease likely represents behaviors such as foraging, drinking, or movement among hibernacula. We also examined the effects of Pd status (positive or negative), environmental conditions (ambient temperature and precipitation) and stage of the hibernation period. We hypothesized that nighttime activity would vary with hibernaculum type, *Pd* status, and environmental conditions. We predicted that activity outside the hibernaculum would be greater in aboveground hibernacula because bats would be more responsive to outside environmental conditions and that bats in *Pd*-negative sites would be more active than those in *Pd*-positive sites because bats infected with *Pd *need to conserve energy^40^. We also predicted that activity would be positively associated with ambient temperature and negatively associated with nighttime precipitation and be highest during the early and late parts of the hibernation season.

## Methods

### Study sites

We monitored nighttime activity at 13 sites across Alabama, Florida, Georgia, and South Carolina, USA over two winters although one site (Bullock Creek Culvert, Alabama), was only monitored during 2021–22 (Fig. 1). Eight of the sites were aboveground structures (six culverts and two bridges) and five were subterranean (two caves, two incomplete railroad tunnels, and one abandoned gold mine; Table 1). Prior to and during the study, all sites were monitored for the presence of *Pd* and WNS using standard protocols as outlined by the USGS National Wildlife Health Center (NWHC; https://d9-wret.s3.us-west-2.amazonaws.com/assets/palladium/production/s3fs-public/media/files/NWHC%20Winter%202023-2024%20Bat%20Submission%20Guide_v01022024.pdf). Samples taken prior to our study were analyzed by various members of the White-Nose Syndrome Diagnostic Laboratory Network, and samples taken during this study were processed by the laboratory at Kennesaw State University, one of the members of the Network. Following the guidelines of the NWHC, we considered a site to be *Pd-*positive if at least some samples had a Ct ≤ 37. *Pd* was documented in the four *Pd-*positive subterranean sites and three of the four aboveground *Pd*-positive sites prior to the onset of the study (Table 1). One site was first tested in the first year of our study and was *Pd*-positive. However, none of the bats in the aboveground *Pd-*positive sites showed signs of WNS. One of the sites, Black Diamond Tunnel in northwestern Georgia received an anti-fungal treatment from 2016 to 2023^41^. However, a high proportion of bats tested in this hibernaculum still had Ct < 37 during this study^42^. Due to the progression of the disease from its origin in New York (https://whitenosesyndrome.org/where-is-wns), *Pd*-positive sites were in the northern and higher elevation portions of the study area, whereas *Pd*-negative sites were in the southern and lower elevation portions (Fig. 1). The number of tricolored bats inhabiting the sites ranged from 3 to 654 (Table 1).

**Table 1:** Type, latitude, and presence or absence of *Pseudogymnoascus destructans* (*Pd*), at each of the structures monitored for tricolored bat (*Perimyotis subflavus*) acoustic activity during winters 2020–21 and 2021–22. Also presented are the number of tricolored bats in the structure each winter, the number of nights during which activity was monitored at each structure, mean ± 1 s.d. number of call files per night, and percent of nights during which tricolored bat activity was detected. If more than one census was taken at a site during a winter, the number of tricolored bats presented represents the mean.

Hibernacula Type	Structure Type	Latitude	Pd Status (Year Positive)	Number Tricolored Bats 2020–21	Number Tricolored Bats 2021–22	2020–21 Detector Nights	2021–22 Detector Nights	2020–21 Acoustic Activity (Mean Files/ % Nights)	2021–22 Acoustic Activity (Mean Files/ % Nights)
Aboveground	Bridge	33.279	Negative	3	3	161	230	4.1 ± 11.9 46.1%	2.8 ± 7.9 37.3%
Aboveground	Bridge	33.309	Negative	3	3	153	289	39.6 ± 99.9 60.2%	31.0 ± 50.2 80.7%
Aboveground	Culvert	31.980	Negative		82		199		8.7 ± 19.4 67.2%
Aboveground	Culvert	30.182	Negative	9	2	169	199	4.0 ± 9.0 83.5%	2.5 ± 4.0 68.7%
Aboveground	Culvert	33.692	Positive 2019 - 20	189	210	188	225	2.0 ± 5.3 30.9%	13.3 ± 45.6 67.8%
Aboveground	Culvert	33.611	Positive 2020–21	31	24	73	106	0.42 ± 2.36 5.5%	0.5 ± 1.48 20.8%
Aboveground	Culvert	33.087	Positive 2019 - 20	53	49	103	116	2.1 ± 6.8 29.1%	11.4 ± 22.7 71.6%
Aboveground	Culvert	33.618	Positive 2019 - 20	107	150	158	116	0.08 ± 0.32 6.6%	1.2 ± 2.3 35.0%
Subterranean	Cave	29.716	Negative	26	27	88	148	2.8 ± 2.4 80.7%	3.4 ± 3.8 79.1%
Subterranean	Tunnel	34.878	Positive 2013 - 14	272	364	126	112	1.5 ± 6.6 22.2%	0.8 ± 1.8 34.8%
Subterranean	Tunnel	34.811	Positive 2013 - 14	63	87	128	165	1.51 ± 5.2 20.3%	0.9 ± 2.29 29.1%
Subterranean	Mine	34.630	Positive 2014 - 15	5	8	47	133	0.35 ± 1.2 10.9%	0.05 ± 0.26 4.5%
Subterranean	Cave	33.742	Positive 2015 - 16	654	532^a^	150	160	1.7 ± 4.8 40.9%	15.9 ± 24.8 73.9%

^a^No census was conducted in 2021–22. This is an estimate based on the mean of the three prior censuses.

### Acoustic sampling and analysis

Because tricolored bats in the northern hibernacula in our study area enter hibernation in late September or early October and emerge in early to mid-March^43^, our goal was to sample October through March. However, sampling started later in 2020–21 due to delays related to the SARS-COV2 pandemic and we were only able to monitor bat activity at each site from late November or early December 2020 through mid- to late March 2021. In the second year we were able to monitor activity from early October 2021 through mid- to late March 2022. We placed Anabat Express bat detectors (Titley Scientific, Columbia, MO) at the entrance of each hibernaculum; when there were two entrances such as in the case of culverts, bridges, and one cave, we placed a detector at each entrance. Detectors were placed on trees or poles approximately 2–3 m above ground and approximately 5–20 m from the entrance depending on the hibernaculum. Although some activity can occur during the day in WNS-affected bats^5^, most studies have shown that activity of bats during winter occurs primarily within the early part of the night^44–46^. Thus, we programmed detectors to run from 1800 to 2400 h each night.

We used Kaleidoscope Pro version 5.4.3 (Wildlife Acoustics, Maynard, MA) to identify call files to species. We modified the potential species lists for each site so that only species that were likely to be in the area based on range maps were included for each site. To minimize misclassification, we used the Conservative setting and required at least five pulses be present. We did not manually vet all tricolored bat call files but spot-checked files from each site to verify correct identifications, particularly on nights when high numbers of tricolored bat calls were recorded. In most cases we deemed that the identifications were correct. In those few cases where we did not agree with the auto-identifications, we did not include those files in the analyses. For those sites where two detectors were placed, we averaged the nightly call files from the two detectors.

### Weather data

We obtained minimum, maximum, and mean daily temperatures and hourly precipitation data from the nearest weather station to each site. Data were obtained either from Remote Automated Weather Stations (RAWS; https://raws.dri.edu/) or through Mesowest (https://mesowest.utah.edu/) when RAWS stations were farther away. We averaged hourly temperatures to obtain daily mean temperature and totaled the rainfall for 1700–2400 h (i.e., the recording period plus the hour prior).

### Statistical analysis

We used R version 4.4.0 for all analyses. We tested for collinearity among our independent variables and found that mean, maximum, and minimum temperatures were highly correlated (*r* ≥ 0.90). Therefore, we only used mean daily temperature in our analyses.

We used linear mixed models (Package lme4)^47^ with site as the random effect to test for differences in temperature and precipitation between years using the full dataset (October–March) and December–March data only due to the shortened field season in 2020–21. We also used a chi-square analysis (Package gmodels; https://cran.r-project.org/web/packages/gmodels/index.html) to test whether the proportion of nights during which rainfall occurred differed between years.

Because data were collected late-November through March in 2020–21and for the entire winter season (October through March) in 2021–22, we analyzed the two years of acoustic data separately. To account for repeated measures (i.e., multiple nights) at each site as well as Type and Status of each site, we used Site within Type and Status as a random effect in the models which adjusted for the repeated observations within each site. We used generalized mixed models (Package lme4) to test the effects of hibernaculum type (Type), *Pd *status (Status), ambient temperature (Temperature), presence or amount of precipitation (Precipitation), and stage within the hibernation period (Date) on tricolored bat activity. We considered including latitude and elevation as independent variables, but they were synonymous with site. We first tested whether the presence of activity during the night (0 or 1) was affected by these factors using a binomial distribution and then we tested whether the level of activity (number of call files per night) was affected by these factors using a Poisson distribution. Because we averaged the number of calls at sites with two entrances, we used the round function in R to meet the assumptions of a Poisson distribution for those sites where the number of call files per night was averaged. Further, the number of bats in a structure likely influenced the level of activity at a site (i.e., the number of call files was likely related to the number of bats in a site). Therefore, we used the number of bats in each hibernaculum as an offset in our activity models. Tricolored bats enter hibernation in late September at our northern sites^43^, so we used days from October 1 (Date) as our measure of hibernation stage and also included its quadratic term (Date^2^) to account for the differences between the onset and end of hibernation and its middle. Because our goal was to examine the effects of hibernaculum type and *Pd* status on tricolored bat activity as well as the interactions of temperature, period of the hibernation period, and precipitation with hibernaculum type and *Pd* status, we built one model for each year and response type (presence or level of activity) to test these effects. Unfortunately, some interactions were not estimable and could not be included in some models. When this occurred, we included those interactions that allowed us to look at the most interactions. Further, using the amount of precipitation per night resulted in non-convergence of our binomial models so we used the presence of rain during the night as our Precipitation variable in those models. We scaled all continuous independent variables to a mean of 0 and a standard deviation of 1 before analysis and used α ≤ 0.05 to denote significant effects.

## Results

We monitored bat activity over 3,742 detector nights: 539 at subterranean hibernacula and 1,005 at aboveground hibernacula in 2020–21 and 718 at subterranean hibernacula and 1,480 at aboveground hibernacula in 2021–22. We recorded 204,145 files containing bat calls of which 27,007 were identified as tricolored bat calls (946 at subterranean sites and 6,066 at aboveground sites in 2020–21, and 3,631 at subterranean sites and 16,364 at aboveground sites in 2021–22). Other species identified were big brown bats, eastern red bats/Seminole bats (*Lasiurus borealis* or *L*. *seminolus*), hoary bats (*L. cinereus*), silver-haired bats (*Lasionycteris noctivagans*), southeastern myotis, gray bats (*M. grisescens*), eastern small-footed bats (*M. leibii*), little brown bats, northern long-eared bats, evening bats (*Nycticeius humeralis*), and Brazilian free-tailed bats (*Tadarida brasiliensis*). Tricolored bats were active during some nights at all hibernacula with the greatest level of activity (i.e., mean number of files/night) occurring at one of the bridges (Table 1). The proportion of nights that bats were active, as well as the level of activity, varied considerably among sites and between years at some hibernacula but not at others (Table 1).

The number of nights during which rain occurred did not vary significantly between years when data for all months (October through March) were considered (χ^2^ = 2.06, d.f. = 1, *P* = 0.15) or just December through March (χ^2^ = 0.04, d.f. = 1, *P* = 0.84). Further, the amount of rain that fell did not differ between years when all months were considered (*F* = 1.15, d.f. = 1, 2555, *P* = 0.28) or when only December through March were considered (*F* = 2.61, d.f. = 1, 1864, *P* = 0.11). In contrast, temperatures were significantly warmer in 2021–22 than in 2020–21 when all months were considered and for December–March only (*F* = 159.59, d.f. = 1, 2800, *P* < 0.0001 and *F* = 38.15, d.f. = 1, 2133, *P* < 0.0001, respectively). Mean daily temperatures were 9.6 ± 0.58 °C in December–March 2020–2021. In 2021–22, mean daily temperatures were 12.2 ± 0.50 °C from October through March and 11.0 ± 0.58 °C from December through March.

**Fig. 2:**
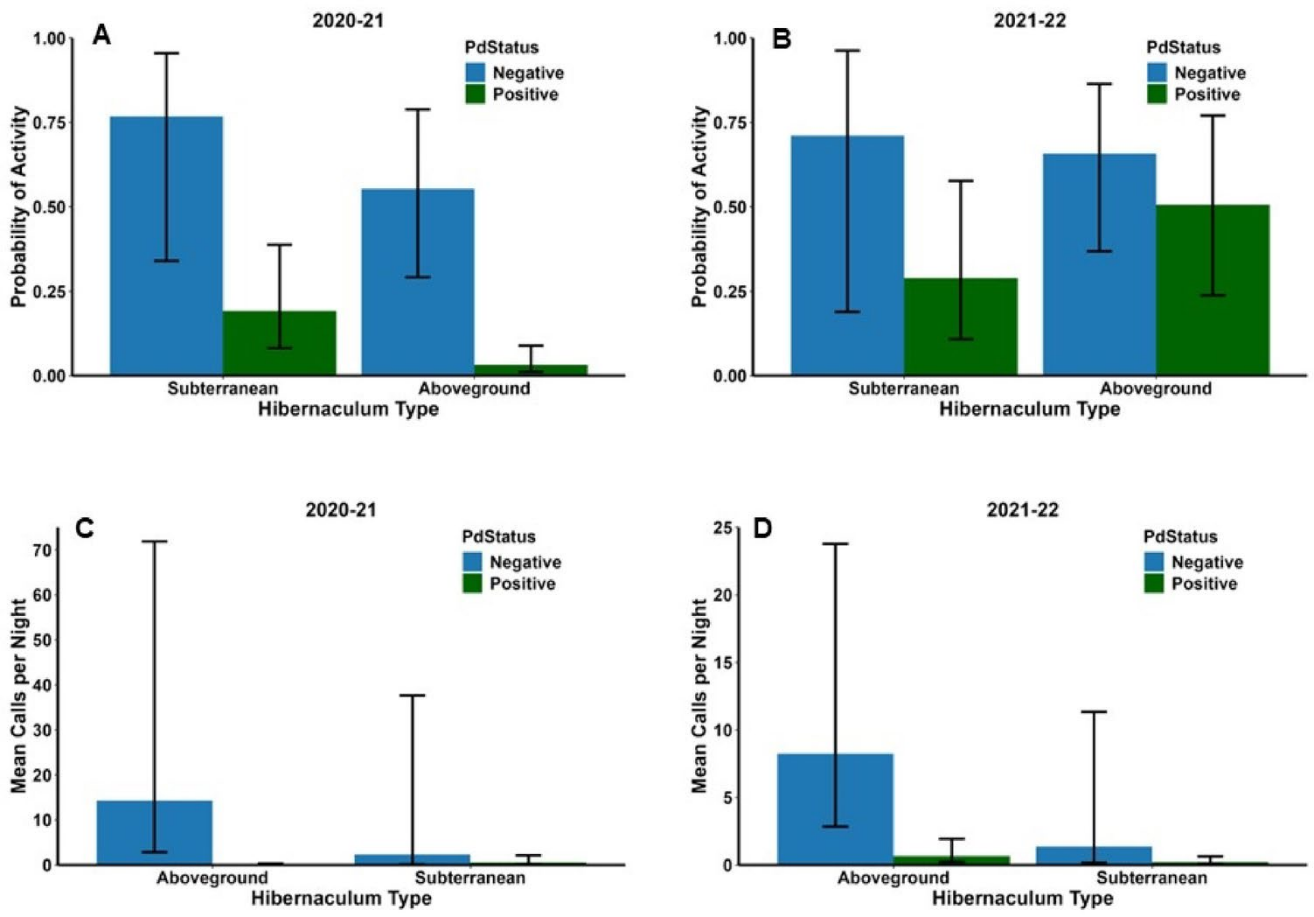
Mean (± 1 S.E.) probability of tricolored bat (*Perimyotis subflavus*) activity (**A** and **B**) and mean (± 1 S.E.) number of calls per night (**C** and **D**) in aboveground and subterranean hibernacula in the southeastern USA during winter 2020–21 and 2021–22.

**Fig. 3:**
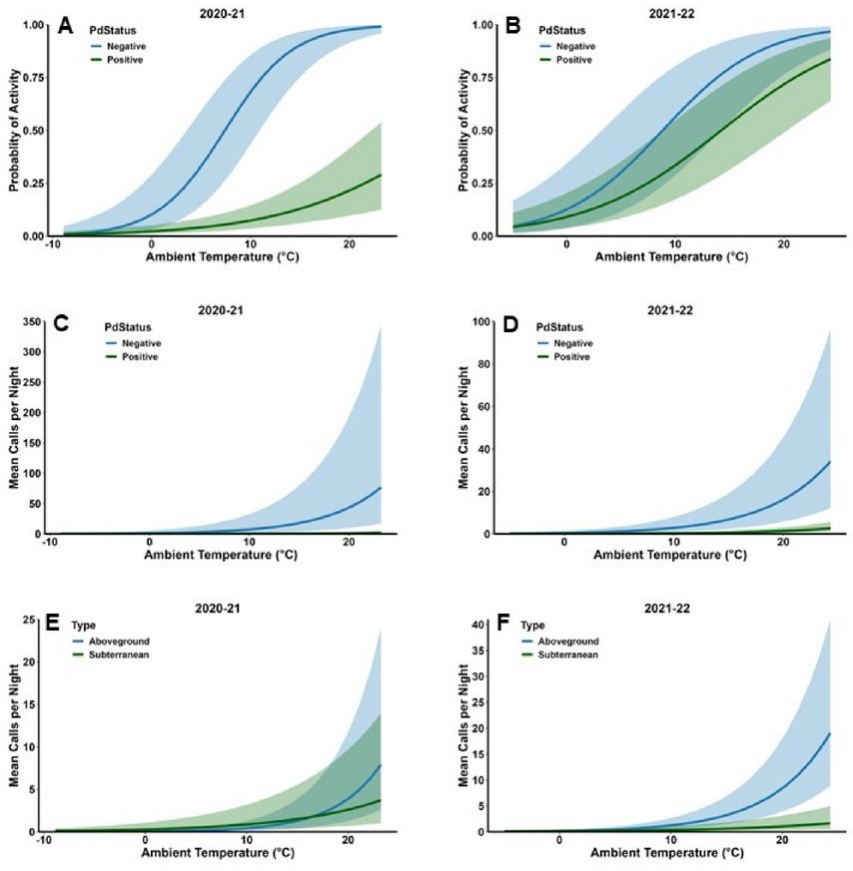
Mean number of calls per night in aboveground and subterranean hibernacula in the southeastern USA in relation to ambient temperature and hibernacula type (**A** and **C**) and *Pd* status (**B** and **D**) during winter 2020–21 and 2021–22. Shaded areas represent 95% confidence limits.

**Fig. 4:**
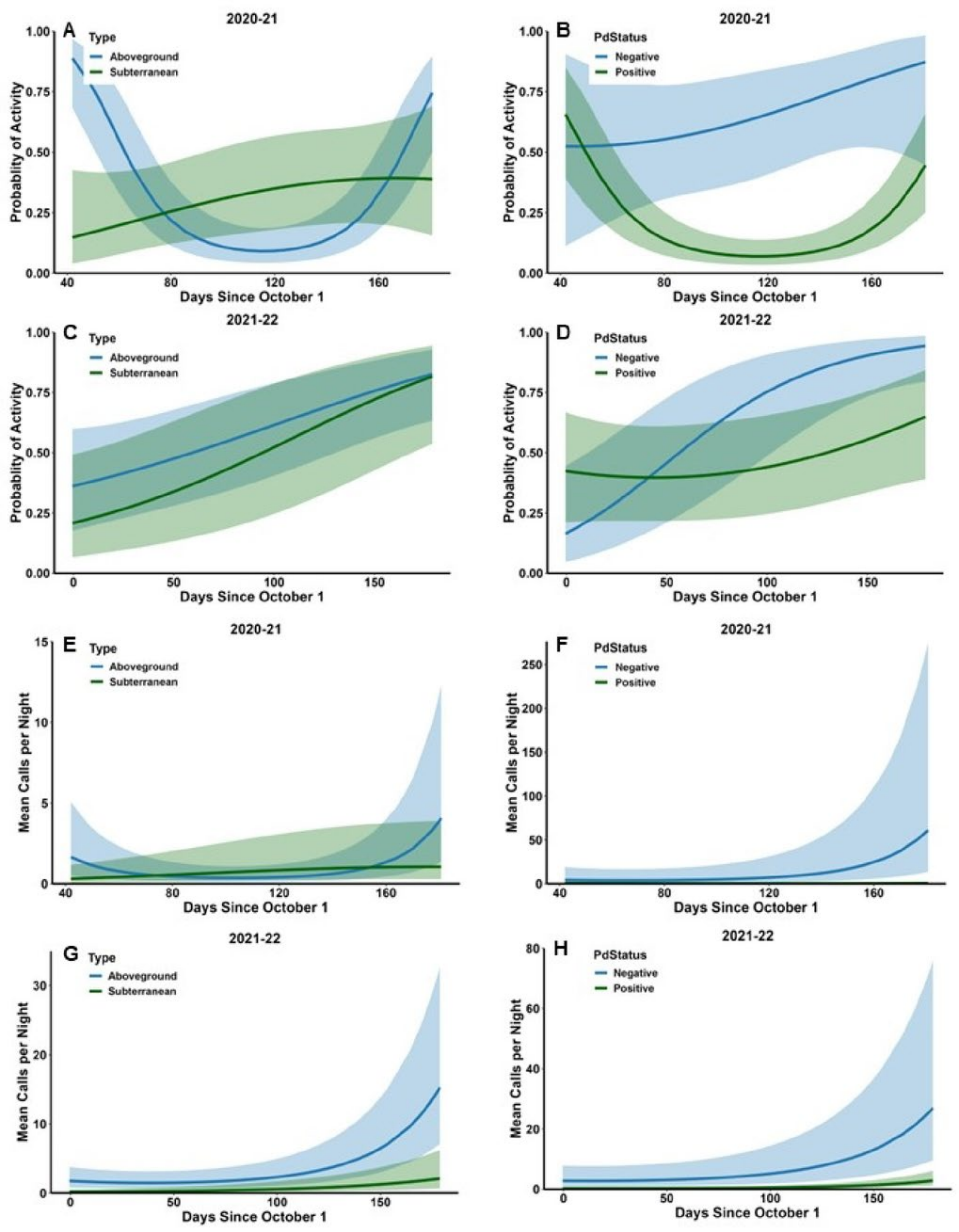
Probability of tricolored bat (*Perimyotis subflavus*) activity (**A**-**D**) and mean number of calls per night (**E**-**H**) in aboveground and subterranean hibernacula in the southeastern USA in relation to stage of hibernation (days since October 1) and hibernacula type and *Pd* status during winter 2020–21 and 2021–22. Shaded areas represent 95% confidence limits.

 Probability of activity did not vary with Type in either year but varied with Status in 2020–21 (Table 2); the interaction between Type and Status was not significant in either year. The probability of activity was significantly greater in *Pd*-negative sites (0.65 ± 0.12) than *Pd*-positive sites (0.07 ± 0.03) in 2020–21 (Fig. 2 A). In 2021–22, probability of activity was 0.68 ± 0.13 in *Pd*-negative sites and 0.42 ± 0.11 in *Pd*-positive sites (Fig. 2B). Temperature, Precipitation, and Date were significant factors in both years (Table 2). Probability of activity was positively related to temperature and days since the onset of hibernation, whereas the relationship between probability of activity and rain during the night was negative (Table 2). However, ambient temperature and days since the onset of hibernation sometimes interacted with Type and Status making interpretation more complex. In 2020–21, the interaction between Temperature and Status was significant, and the interactions between the quadratic term of Date and Type and quadratic term of Date and Status were significant (Table 2). The probability of activity increased with ambient temperature in both *Pd*-negative and *Pd*-positive sites but increased at a greater rate in *Pd*-negative sites than *Pd*-positive sites (Fig. 3 A). The probability of activity increased linearly with Date in subterranean sites and in *Pd-*negative sites but was greater at the beginning and end of the hibernation period in aboveground sites and *Pd*-positive sites (Fig. 4 A and B). In 2021–22, the interaction between Status and Date was significant (Table 2). The probability of activity increased at a greater rate in *Pd*-negative sites as time since the onset of hibernation progressed (Fig. 4D). In 2021–22 the effects of Precipitation did not vary with either Type or Status (Table 2); these interactions could not be tested for 2020–21. Probability of activity was lower during nights with rain in both years (0.09 ± 0.03 in 2020–21 and 0.35 ± 0.09 in 2021–22) versus no rain (0.19 ± 0.05 in 2020–2021 and 0.57 ± 0.09) in both hibernaculum types and *Pd*-positive and *Pd*-negative sites.

**Table 2:** Results of mixed binomial models predicting probability of winter activity for tricolored bats (*Perimyotis subflavus*) during 2020–21 and 2021–22 in relation to hibernaculum type (Type; aboveground or subterranean), presence of *Pseudogymnoascus destructans*in the hibernaculum (Status; positive or negative), mean daily temperature (Temperature), days into the hibernation period and its quadratic form (Date and Date^2^), and the presence or absence of precipitation during the night (Precipitation).

	2020–21				2021–22			
	Estimate	Std. Err.	z	*P*	Estimate	Std. Err.	z	*P*
Intercept	1.358	0.952	1.427	0.154	1.09	1.2	0.908	0.364
Type (Aboveground)	− 0.983	1.089	− 0.903	0.366	− 0.286	1.335	− 0.214	0.83
Status (Positive)	− 2.637	1.067	− 2.47	0.013	− 1.878	1.349	− 1.392	0.164
Temperature	1.547	0.219	7.058	< 0.00001	1.256	0.156	8.046	< 0.00001
Date	0.591	0.251	2.351	0.019	1.365	0.18	7.592	< 0.00001
Date^2^	− 0.502	0.312	− 1.61	0.107	− 0.09	0.149	− 0.6042	0.546
Precipitation	− 0.878	0.235	− 3.728	0.0002	− 1.389	0.4	− 3.471	0.0005
Type*Status	− 0.986	1.291	− 0.763	0.445	1.155	1.58	0.731	0.465
Status*Temperature	− 0.894	0.255	− 3.5109	0.0004	− 0.343	0.192	− 1.785	0.0742
Status*Date	− 0.442	0.242	− 1.8297	0.067	− 1.025	0.172	− 5.962	< 0.00001
Status*Date^2^	0.577	0.299	1.93164	0.053	0.25	0.185	1.353	0.176
Type*Date	− 0.148	0.168	− 0.8796	0.379	− 0.2	0.145	− 1.365	0.172
Type*Date^2^	1.067	0.207	5.16581	< 0.00001				
Status*Precipitation					0.488	0.372	1.312	0.189
Type*Precipitation					0.223	0.358	0.624	0.533

**Fig. 5:**
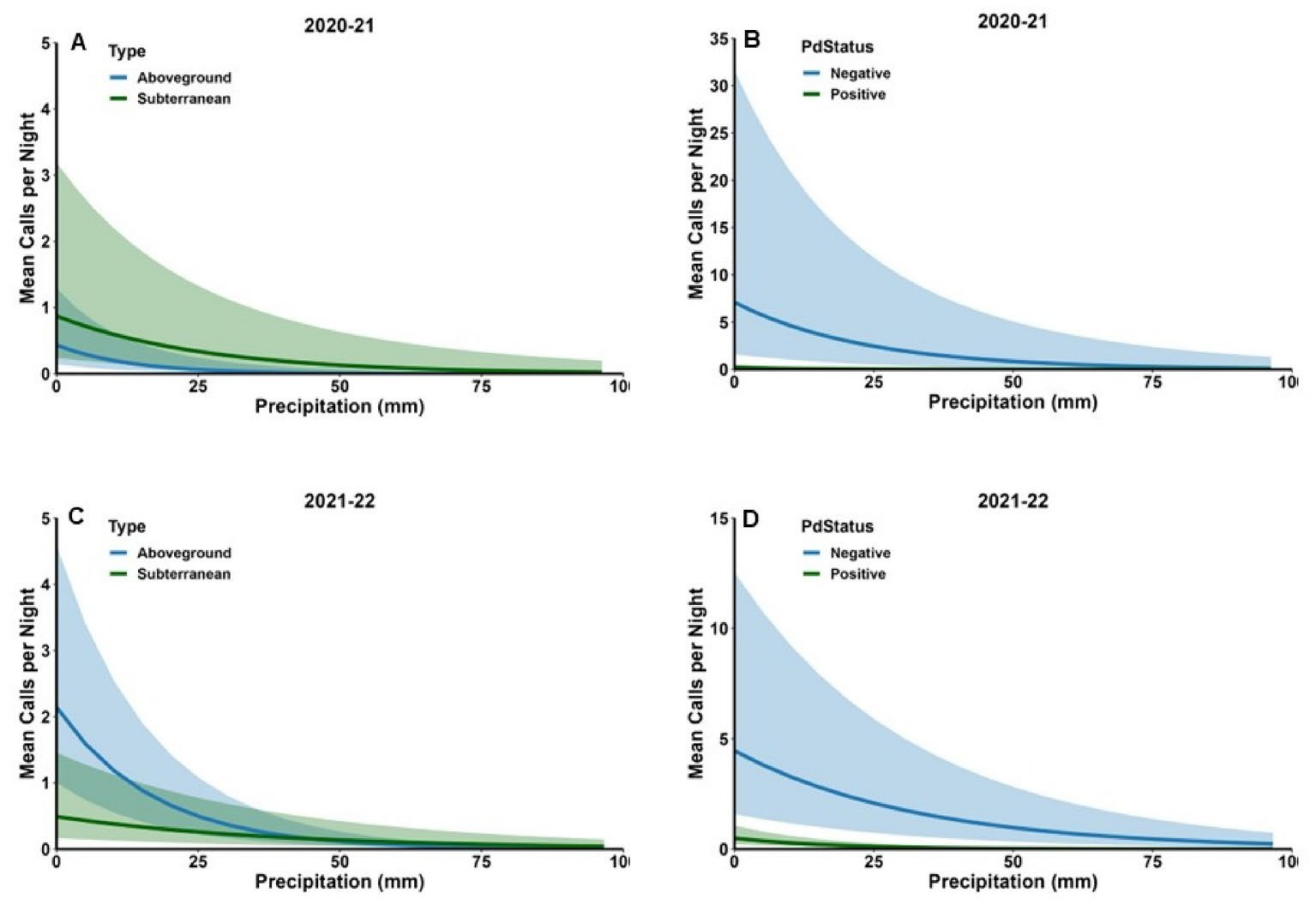
Mean number of calls per night in relation to the amount of precipitation during the night in aboveground and subterranean hibernacula in the southeastern USA during winter 2020–21 and 2021–22. Shaded areas represent 95% confidence limits.

Neither Type nor Status were significant predictors of activity level in either year (Table 3). However, the interaction between Type and Status was significant in 2020–21 (Table 3). The difference between activity levels in *Pd*-negative and *Pd*-positive sites was greater in aboveground hibernacula (14.2 ± 11.8 calls/night versus 0.08 ± 0.06 calls/night) than in subterranean hibernacula (2.3 ± 3.3 versus 0.5 ± 0.4; Fig. 2 C). Activity levels were positively associated with Temperature and Date in both years and negatively associated with Precipitation (Table 3). However, most interactions between these factors and Type and Status were significant. Activity increased at a greater rate with Temperature and Date in *Pd*-negative sites than *Pd*-positive sites in 2021–22 and 2020–21 respectively (Figs. 3 C and D and 4 F H) and decreased more rapidly with Precipitation in 2021–22 (Fig. 5D); this relationship was not significant in 2020–21 but followed a similar pattern (Fig. 5B). In both years, activity increased more rapidly with Temperature (Fig. 3E and G) and Date (Fig. 4E and G) in aboveground hibernacula than subterranean hibernacula. In contrast, activity decreased more rapidly in both years with Precipitation in aboveground sites than subterranean sites (Fig. 5 A and C).

**Table 3:** Results of mixed Poisson models predicting mean number of calls for tricolored bats (*Perimyotis subflavus*) during winters of 2020–21 and 2021–22 in relation to hibernaculum type (Type; aboveground or subterranean), presence of *Pseudogymnoascus destructans*in the hibernaculum (Status; positive or negative), mean daily temperature (Temperature), days into the hibernation period and its quadratic form (Date and Date^2^), and the presence or absence of precipitation during the night (Precipitation).

	2020–21				2021–22			
	Estimate	Std. Err.	z	*P*	Estimate	Std. Err.	z	*P*
Intercept	− 2.967	1.424	− 2.084	0.037	− 3.168	1.081	− 2.932	0.003
Type (Aboveground)	1.823	1.643	1.11	0.267	1.801	1.208	1.491	0.136
Status (Positive)	− 1.478	1.593	− 0.928	0.353	− 1.845	1.211	− 1.524	0.128
Temperature	0.599	0.062	9.743	< 0.00001	0.691	0.038	18.138	< 0.00001
Date	0.689	0.056	12.255	< 0.00001	0.678	0.028	23.851	< 0.00001
Date^2^	− 0.082	0.042	− 1.931	0.053	0.062	0.034	1.806	0.071
Precipitation	− 0.127	0.045	− 2.851	0.004	− 0.2	0.069	− 2.91	0.004
Type*Status	− 3.715	1.929	− 1.926	0.054	− 0.666	1.432	− 0.465	0.642
Status*Temperature	− 0.032	0.055	− 0.579	0.563	− 0.148	0.027	− 5.571	< 0.00001
Status*Date	− 0.594	0.046	− 12.937	< 0.00001	0.002	0.018	0.095	0.925
Type*Date	0.146	0.053	2.76	0.006	− 0.137	0.028	− 4.959	< 0.00001
Type*Date^2^	0.572	0.049	11.594	< 0.00001	0.231	0.036	6.393	< 0.00001
Type*Temperature	0.58	0.061	9.464	< 0.00001	0.49	0.035	13.816	< 0.00001
Type*Precipitation	− 0.252	0.108	− 2.335	0.019	− 0.656	0.09	− 7.282	< 0.00001
Status*Precipitation	− 0.171	0.091	− 1.873	0.061	− 0.541	0.102	− 5.304	< 0.00001

## Discussion

Tricolored bats were active throughout the winter across our 13 sites, similar to other studies conducted on winter bat activity in the south^27,48,49^. However, the probability of tricolored bat activity and the level of activity varied considerably across sites. We found environmental factors and stage of the hibernation season were more important predictors of this variation than hibernaculum type or *Pd* status, likely due to bats’ having evolved their torpor and activity patterns to environmental conditions. However, bats in aboveground and subterranean structures and in structures that were either *Pd-*positive or *Pd*-negative, responded to these environmental factors in somewhat different ways. This suggests that even though hibernaculum type and *Pd* status were not significant factors alone, these factors may contribute to tricolored bats’ winter activity outside hibernacula and thus, their susceptibility to the effects of WNS. For example, although tricolored bats in Stumphouse and Black Diamond Tunnels, both subterranean *Pd*-positive sites, experienced high mortality after the arrival of *Pd*^41,50^, no evidence of mortality and only one incidence of diagnostic signs of WNS have been found in *Pd*-positive culverts (Katrina Morris, Georgia Department of Natural Resources, *pers. comm*.). While we were not able to include latitude or elevation in our models, these factors may have also contributed to our results due to their influence on ambient temperature.

As we predicted, ambient temperature was a strong predictor of both the probability of bat activity on a given night as well as the level of activity (i.e., number of call files), and both the probability of activity and the level of activity increased with ambient temperature. Almost all other studies examining bat winter activity have found a similar relationship between bat activity and temperature^5,29,44,51^. Although there were a few instances of tricolored bat activity occurring when average daily temperatures were < 0 °C, the probability of activity began to increase between approximately 5 °C and 10 °C; the level of activity began to increase when average daily temperatures ranged between 10 °C and 20 °C (Fig. 3). In managed pine stands of east Texas and Louisiana in the Southern Coastal Plains, tricolored bats were not detected at temperatures < 4.5 °C and had a mean threshold temperature for activity of 13.7 °C^52^. This threshold was relatively high compared to other species in the area, suggesting that tricolored bats are more likely to stay in torpor than other species, even on warmer nights. Tricolored bats are more active away from their roosts when nighttime temperatures rise above 10 °C^28^, which may be related to increased availability of insects^53^, decreased energetic costs of flight at warmer temperatures^54^, or both. Further, bats roosting in bridges and tree foliage are more likely to be active than bats roosting in more thermally stable tree cavities^28^. The highest activity that we observed in the current study was for bats in one of these bridges.

Although activity increased with temperature in both above- and belowground sites and at *Pd*-positive and *Pd*-negative sites, there were some differences in the rates of those increases. For example, probability of activity increased far more rapidly with temperature in *Pd*-negative sites than *Pd*-positive sites in 2020–21 (Fig. 3 A), and the level of activity increased at a greater rate in *Pd*-negative sites in that year (Fig. 3C). Similarly, the level of activity increased more rapidly with temperature in aboveground hibernacula than subterranean ones in both years. Tricolored bats in aboveground hibernacula are more likely to arouse near dusk^28^, whereas tricolored bats in subterranean sites arouse randomly throughout the day^37^. Thus, tricolored bats in aboveground sites may be better able to assess nighttime ambient conditions and respond more quickly when conditions are favorable for outside activity. Bats in *Pd*-positive sites may need to conserve more energy than those in *Pd*-negative sites due to the effects of the disease^40^, and therefore, may be less likely to leave the roost unless conditions are highly favorable^55^.

Tricolored bat activity was negatively associated with the presence and amount of precipitation during the night. Further, the decrease in activity with rainfall was steeper in aboveground hibernacula and *Pd*-negative sites. Few studies have examined the effects of precipitation on bat activity; however, in Portugal winter bat activity was negatively associated with precipitation^56^. Torpor bout lengths of tricolored bats using aboveground roosts in winter are positively associated with precipitation in South Carolina^28^, and the probability of summer torpor in little brown bats increases with the presence of rain^57^. When rain results in wet pelage and subsequent increased evaporative cooling, energetic costs increase significantly possibly due to increased metabolic rates^58^. Wet fur may also affect flight dynamics and rain may affect sensory capabilities such as echolocation. All these factors may contribute to the negative association of outside bat activity with rain. The more rapid shutdown of activity by tricolored bats in aboveground hibernacula in response to rain may be due to their increased ability to monitor environmental conditions such as the presence of rain due to the more exposed nature of the roost^59^. In contrast, bats in subterranean hibernacula may need to exit the site to assess conditions.

We predicted that activity to be greatest at the beginning and end of the hibernation season, as observed in other bat communities^29,60,61^. However, this response only occurred for the probability of activity in 2020–21 and only in aboveground structures and at *Pd*-positive sites (Fig. 4). Instead, activity tended to increase as the hibernation period progressed. The presence of a curvilinear response in 2020–21 and not in 2021–22 is particularly curious since we did not start monitoring activity until late November or early December in 2020–21, after the period that we predicted that activity would be high. Increased activity at the end of the season is likely related to bats starting to emerge from hibernation^43^. Low activity at the beginning of the season may reflect bats entering the hibernacula to begin hibernation for the winter. For example, two tricolored bats that were radio-tagged outside Stumphouse Tunnel (one of the sites in this study) in late September-early October roosted in trees for a few days and then entered the tunnel and did not leave for over a month^62^. One of these bats was observed in the tunnel throughout the winter. Outside activity of tricolored bats was also minimal between mid-October and February or March at two caves in Indiana^63,64^. We observed that tricolored bat activity increased at a greater rate at the end of the season in aboveground hibernacula and *Pd*-negative sites. Tricolored bats in aboveground hibernacula may be more responsive to ambient conditions and may leave the hibernacula earlier to move to summer habitats than those in subterranean structures although, we are unaware of any studies that have examined this. Little brown bats with WNS choose roosts that allow them to use daily torpor after emergence from hibernacula to conserve energy^65^. Similarly, tricolored bats in *Pd*-positive sites in our study may have stayed in hibernacula longer to conserve energy, resulting in lower activity at the end of hibernation season compared to bats in *Pd*-negative sites.

Winter temperatures are projected to increase over the next several decades in the southeast^66^, which suggests that activity of tricolored bats outside hibernacula may also increase, particularly in aboveground hibernacula. However, more frequent arousals and activity outside hibernacula may also lead to increased predation, increased risk of fat depletion and dehydration, and phenological mismatches such as lack of insect availability during activity periods, particularly for bats that hibernate in subterranean hibernacula and more northern latitudes^9^. For example, tricolored bats that hibernate in caves in Florida, which has a subtropical climate, have declined by 70% in the absence of WNS^67^. The decline is closely related to increasing temperatures and may be due to increased energy use related to hibernating at warmer temperatures. Therefore, even though outside activity may be higher in warmer winters, potentially helping to mitigate the effects of WNS, increasingly warm winters may have long-term effects on tricolored bat populations.

Once *Pd *and WNS were introduced to North America in New York it spread north, south, and west^12^with the spread south occurring primarily down the Appalachian Mountains^68^. Once *Pd* arrives in an area it usually becomes widespread (https://whitenosesyndrome.org/where-is-wns). Hence, it was often not possible to find *Pd*-negative sites near *Pd*-positive sites. Consequently, three of our five *Pd*-negative sites were considerably farther south than our *Pd*-positive sites (Fig. 1). Thus, some of our results related to the effects of *Pd* status may have been related to geographical effects. While we were not able to account for all of these effects, we did account for temperature which is often related to latitude and elevation. We were also only able to include one belowground *Pd*-negative site in our study which may have affected our ability to detect the effects of hibernaculum type independent of *Pd* status and environmental conditions. We encourage more research on activity of tricolored bats as well as other species in *Pd*-negative belowground hibernacula to better assess their susceptibility to WNS should *Pd* arrive.

Our results suggest that tricolored bats using aboveground hibernacula such as culverts or bridges, may be less susceptible to the effects of WNS compared to those using subterranean sites due to increased activity and, in some areas, may not even be hibernating^48^. However, use of aboveground hibernacula may have some costs. For example, temperatures within aboveground hibernacula are far more variable than those in subterranean hibernacula^42^. Thus, bats using these structures may be subject to freezing events^69^. These bats may also be subject to greater predation rates because the sites are usually smaller and more easily accessible than subterranean sites^70^. Further, noise associated with traffic above bridges and culverts may decrease bats’ foraging efficiency^71,72^. Finally, the stream and river systems associated with bridges and culverts and used by bats as travel and foraging corridors, may also serve as a transmission corridor for *Pd*^36^. In the southeastern USA, this may result in *Pd* being transmitted to caves in karst regions of Florida and coastal Alabama that are currently *Pd *free. Tricolored bats in these caves will likely be susceptible to similar morbidity and mortality from WNS as tricolored bats in northwestern South Carolina, which have experienced 90% declines, due to similar torpor patterns^37,50^. However, some bats in subtropical caves, such as those in Florida, may leave these caves and move to aboveground hibernacula^67^. Thus, predicting the susceptibility of tricolored bats to the effects of *Pd* and WNS will require far more information on their behavior and torpor patterns during winter across the WNS-free zone.

## Data Availability

Data collected during and analyzed during the current study are available from the correspondingauthor on reasonable request.
